# Development and Optimization of an Airborne Formaldehyde Microfluidic Analytical Device Based on Passive Uptake through a Microporous Tube

**DOI:** 10.3390/mi10120807

**Published:** 2019-11-23

**Authors:** Anaïs Becker, Christina Andrikopoulou, Pierre Bernhardt, Ruben Ocampo-Torres, Claire Trocquet, Stéphane Le Calvé

**Affiliations:** 1 Université de Strasbourg, CNRS, ICPEES UMR 7515, F-67000 Strasbourg, France; ana.becker@unistra.fr (A.B.); c.andrikopoulou@gmail.com (C.A.); ocampo@unistra.fr (R.O.-T.); 2 In’Air Solutions, 25 rue Becquerel 67087 Strasbourg, France; pbern@inairsolutions.fr (P.B.); ctrocquet@inairsolutions.fr (C.T.)

**Keywords:** microdevice, formaldehyde, microfluidics, microporous membrane, indoor air, passive sampling

## Abstract

This paper describes a compact microfluidic analytical device developed for the detection of low airborne formaldehyde concentrations. This microdevice was based on a three-step analysis, i.e., the passive gaseous formaldehyde uptake using a microporous membrane into an acetylacetone solution, the derivatization with acetylacetone to form 3,5-diacetyl-1,4-dihydrolutidine, and the quantification of the latter using fluorescence detection. For a rapid and easier implementation, a cylindrical geometry of the microporous element was considered to perform laboratory-controlled experiments with known formaldehyde concentrations and to establish the proof of concept. This work reports the evaluation of the uptake performance according to the microporous tube length, the liquid flow rate inside the tube, the gas flow rate outside the tube, and the gaseous formaldehyde concentration. A 10.0 cm microporous tube combined with a gas flow rate of 250 NmL/min (normal milliliters per minute) and a liquid flow rate of 17 µL/min were found to be the optimized conditions. In these experimental conditions, the fluorescence signal increased linearly with the gaseous formaldehyde concentration in the range 0–118 µg/m^3^, with the detection limit being estimated as 0.13 µg/m^3^ when considering a signal-to-noise ratio of 3.

## 1. Introduction

Formaldehyde is a major pollutant of indoor air due to its multiple sources (materials, combustion, painting, etc.). Several studies [1,2,3,4] have shown that indoor formaldehyde concentrations are 2–15 times higher than those measured outdoors, and may vary typically between 10 to 100 μg/m^3^. Casset et al. have shown that formaldehyde is implicated in allergic diseases, particularly for asthmatic people [5]. Starting from 2004, formaldehyde is considered as a carcinogenic compound for humans by the International Agency for Research on Cancer [6]. Consequently, current French regulations recommend a limit of 30 μg/m^3^ for chronic exposure and 50 μg/m^3^ for acute exposure [7] in order to reduce the health impact.

The main analytical methods allowing for the quantification of airborne formaldehyde concentrations uses an active or passive sampling on a 2,4-dinitrophenylhydrazine (DNPH) tube for several hours or several days, respectively, followed by an HPLC/UV analysis, which is time-consuming and involves a laboratory treatment with bulky instruments. In addition, this approach does not provide real-time results and only gives an average value of the concentration.

Many alternative methods oriented toward real-time and in-field detection have been developed, such as the Hantzsch monitor, proton-transfer-reaction mass spectrometry (PTR-MS), GC-MS, and infrared diode laser spectroscopy [8,9,10,11]. However, most of these methods rely on bulky apparatuses, and as such, are not adapted for indoor air monitoring where portability is a key point. Among the methods previously developed, some use specific reagents that can react with formaldehyde to produce a compound that can be detected, either via colorimetry or fluorescence [12,13,14]. Miniature and portable systems based on a color change in solid materials, which is conveniently used for the detection, have been developed [15,16]. Nevertheless, autonomic and fully continuous monitoring was not achieved since with time, the solid detection element got saturated and could not be renewed without human intervention.

Even if measuring 30 or 50 μg/m^3^ is not particularly complicated from an analytical point of view, it is more challenging to perform it in real-time by means of a sufficiently precise and rapid apparatus suitable for indoor environments, i.e., small, portable, with a low operation noise, and running on a battery. Most of the near real-time sensitive and precise techniques available on the market run on mains power and are not portable, except for the instruments marketed by Ethera (Nemo), more recently by In’Air Solutions (In’Air µF-1), and probably a few other manufacturers. However, none combines satisfactory portability, a small footprint, battery operation, being silent, a time resolution lower than 1 min, and a detection limit better than 1 µg/m^3^. Usually, the real-time techniques use a gas pump and a mass flow controller to perform the sampling, making them particularly costly, bulky, and energy intensive, which is a strong limitation for a portable instrument.

The main goal of this work was to solve these issues and thus to develop a formaldehyde analytical method and the associated laboratory prototype in order to quantify gaseous formaldehyde concentrations in near-real time. The technique was based on three sequential steps:

- Passive gaseous formaldehyde uptake into an aqueous acetylacetone solution through a microporous membrane;
- The derivatization reaction at 65 °C to quantitatively convert formaldehyde into an easily detectable fluorescent molecule;
- The detection of 3,5-diacetyl-1,4-dihydrolutidine (DDL) using fluorescence.

The gaseous formaldehyde uptake efficiency has been optimized under controlled laboratory conditions by varying the microporous tube length, the liquid flow rate inside the tube, the gas flow rate applied close to the porous interface, and the gaseous formaldehyde concentration. Finally, the linearity and the sensitivity of the microdevice have been evaluated.

## 2. Materials and Methods

### 2.1. Chemicals

The reaction between acetylacetone solution (Fluoral-P) and formaldehyde producing DDL was first investigated via a Hantszch reaction [17] in detail. Acetylacetone solution (0.01 M) was prepared by mixing 0.3 mL of acetic acid (100%, Merck, Molsheim, France), 0.2 mL of acetylacetone (99%, Merck), and 15.4 g of ammonium acetate (98%, Sigma-Aldrich, Lyon, France) in 200 mL Milli-Q water (18.2 MΩ∙cm at 25 °C, Millipore, Molsheim, France). The reagent bottle was equipped with a DNPH cartridge to purify the air entering the vial to avoid any contamination of the reagent with formaldehyde present in the ambient air.

Gaseous formaldehyde mixtures in the range of 11–118 µg/m^3^ (9–96 ppb) were prepared using a homemade source of gaseous formaldehyde. Briefly, this source generated gaseous formaldehyde by applying a controlled gas flow (5–20 NmL/min, where this unit means “normal milliliters per minute”) in a microporous tube immersed in a concentrated formaldehyde solution (0.0925%) regulated at 10 °C using a Peltier module (AA-019-12-22, Alcyon électronique, Beynes, France). This enriched gaseous formaldehyde stream was then diluted with a controlled flow (500–2000 NmL/min, Messer, Suresnes, France) of pure air (>99.999%). The 0.0925% formaldehyde solution was prepared by mixing a commercial formaldehyde solution (37% in water, Sigma-Aldrich) with Milli-Q water (18.2 MΩ∙cm at 25 °C, Millipore). Finally, the generated gaseous formaldehyde concentrations were precisely quantified using the conventional sampling method on DNPH cartridges followed by HPLC/UV analysis (Thermo Finnigan, spectra system, Villebon sur Yvette, France) already described elsewhere [10,18,19,20].

The overall relative uncertainties on the measured gaseous concentrations were in the range 9–11% and were calculated from the uncertainties of the mass flow controllers of the formaldehyde source, the gas sampling, and the HPLC analysis of DNPH cartridges.

### 2.2. Setups and Experimental Conditions

#### 2.2.1. Overall Device Configuration

**Figure 1:**
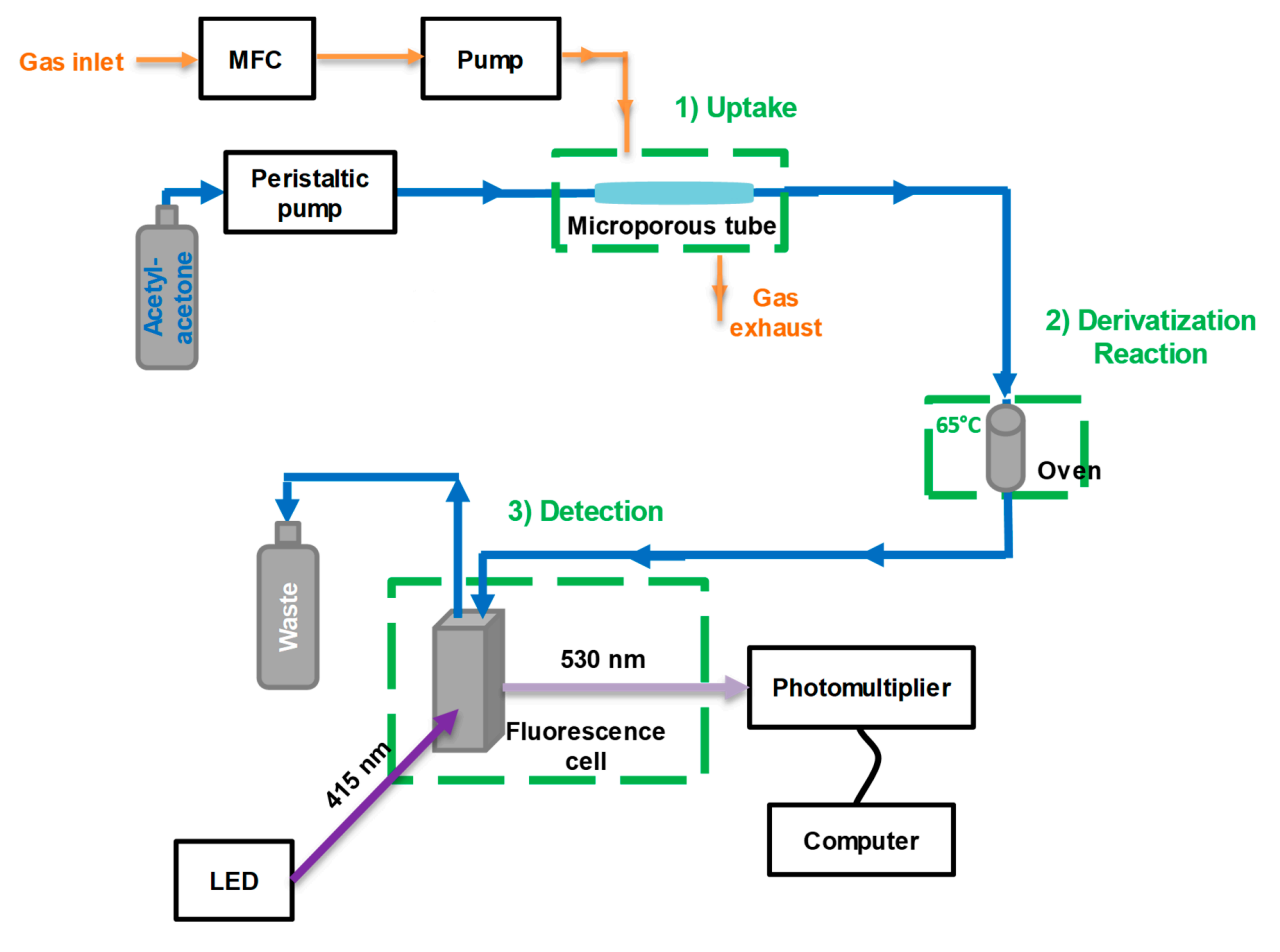
Schematic representation of the setup used for the quantification of gaseous formaldehyde. MFC: Mass Flow Controller.

The overall device proposed for the quantification of gaseous formaldehyde is represented in Figure 1. It consisted of three sequential steps:

- Gaseous formaldehyde uptake into the aqueous acetylacetone solution using a microporous tube;
- Derivatization reaction with acetylacetone at 65 °C;
- DDL detection using fluorescence.

**Figure 2:**
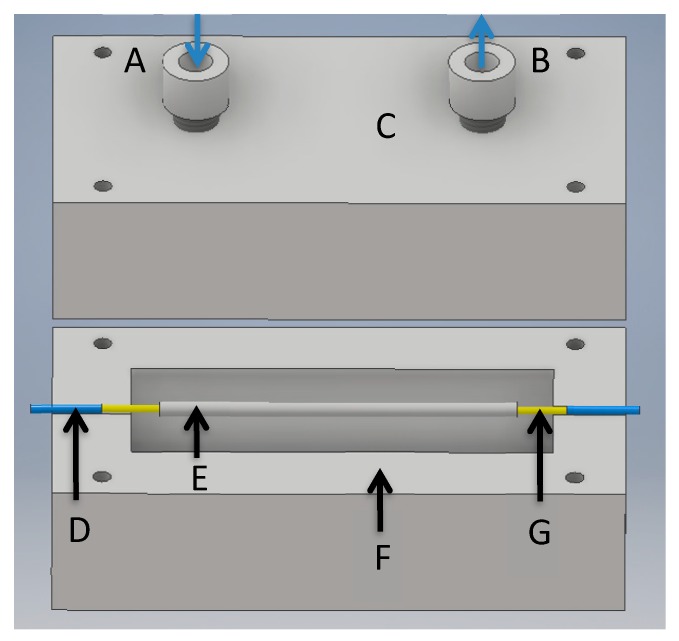
Setup of the microporous tube housing: (**A**) gas flow inlet, (**B**) gas flow outlet, (**C**) peek gas connectors, (**D**) 1/16-inch OD and 250-µm ID tubes, (**E**) microporous tube, (**F**) housing box, and (**G**) needles for microporous tube connection with 1/16-inch OD tubes.

The aqueous solution of acetylacetone (0.01 M) was pumped at a flowrate of 9–33 μL/min thanks to a peristaltic pump (Ismatec, Reglo Lab, Cole-Parmer GmbH, Wertheim, Germany) and conveyed through a 1/16-inch OD and 250-µm ID Teflon tubes (U88511, Interchim, Montluçon, France) all along the device. The liquid solution the flowed inside the hydrophobic polytetrafluoroethylene (PTFE) microporous tube. The microporous tube lengths ranged between 3.2 and 27.5 cm, and two connectors enabled the assembly with the microporous tube (Sumitomo, Paris, France) and the 1/16-inch tube at both the inlet and the outlet (see Figure 2). The microporous tube had a porosity of 60%, an internal diameter of 0.0354 inch (0.90 mm) and an outer diameter of 0.0669 inch (1.70 mm).

In this configuration, since gaseous formaldehyde is very soluble in water according to its high Henry’s law constant of 5000 M/atm^1^ [21], it passes through the porous interface while the liquid remains inside.

Such a device can be directly installed in the environment where gas analysis is required. Indeed, no gas pump is absolutely needed since the uptake of gaseous formaldehyde acts as “passive sampling” [22]. However, a slow gas diffusion compared to the uptake itself could limit the quantity of gaseous formaldehyde available close the microporous interface. A more efficient gas uptake can be promoted by renewing the gas phase near the microporous interface using either a gas pump or a set of adequate fans, directing the gas sample to the porous interface.

The laboratory device was obtained by placing all the components (electronics included) on a plexiglass plate 25 cm long and 15 cm wide. Its weight was about 2.9 kg. It was controlled by a homemade software under Windows which allowed for the control of each element as well as the acquisition of the signal.

#### 2.2.2. Laboratory Validation Setup

Although this device configuration could probably avoid the use of a noisy gas pump and an expensive mass flow controller by using a set of fans coupled to an adapted mechanical design in the future, it appeared to be easier to optimize the set-up using a conventional gas sampling made of a gas pump and a mass flow controller. Therefore, the porous interface was placed in a specifically designed housing, as shown in Figure 2. This chamber, made of acrylonitrile butadiene styrene (ABS) using a 3D printer for rapid prototyping, enabled the isolation of the microporous tube from the ambient atmosphere and the injection of either pure air or known gaseous formaldehyde concentrations at different flow rates in the range 50–300 mL/min.

These gas flow rates were generated and controlled using a mass flow controller (Bronkhorst, 0–500 Nml/min, Montigny-lès-Cormeilles, France) and a pump (KNF LAB type N86KT18, Village-Neuf, France), where a part of the gas stream generated by the gaseous formaldehyde source was injected into the chamber, while the rest was rejected to waste. 

Then, the liquid solution entered a temperature-regulated oven maintained at 65 °C where the reaction with acetylacetone solution was promoted. For the given liquid flow rate of 17 µL/min, the reaction’s time of about 3.5 min was controlled through the length of the tubing installed inside the oven. Finally, the solution entered the fluorescence cell for DDL detection, with the latter being excited by an LED centered at 415 nm and fluorescence being collected on a photomultiplier (H10722-210, Hamamatsu, Massy, France) coupled to a 530 ± 40 nm bandpass filter. The fluorescence signal was then amplified with a gain set to 34–50% and averaged over two seconds. The resulting response time of the analytical prototype depended on the liquid flow rate and the microporous tube length: a higher liquid flow rate and a shorter microporous tube length induced a smaller response time.

## 3. Results and Discussion

The uptake efficiency of the gaseous formaldehyde was evaluated and optimized under laboratory conditions by varying the microporous tube length, the liquid flow rate inside the tube, the gas flow rate injected around the microporous tube housing, and the gaseous formaldehyde concentration. The results are detailed and then discussed in the sections below.

**Figure 3:**
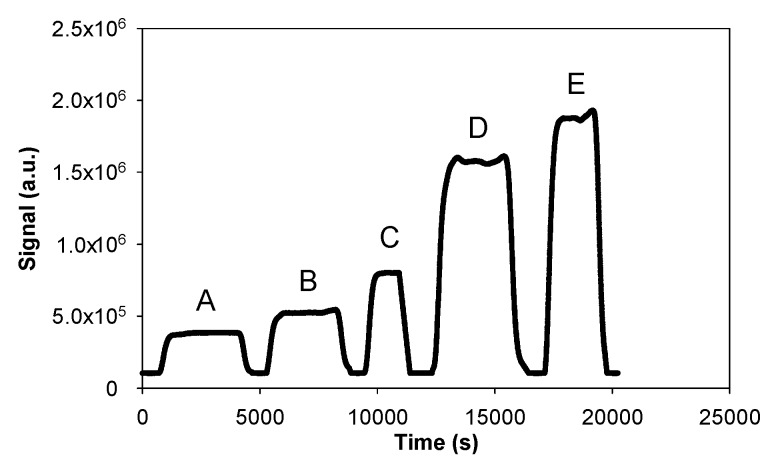
Raw fluorescence signal obtained with a gas flow rate of 250 NmL/min, a liquid flow rate of 17 µL/min, a photomultiplier gain set at 40% (saturation at 2.07 × 10^6^ a.u.), a 10-cm microporous tube length, and various gaseous formaldehyde concentrations: (**A**) 11 µg/m^3^ (9 ppb), (**B**) 23 µg/m^3^ (18.7 ppb), (**C**) 40 µg/m^3^ (33 ppb), (**D**) 85 µg/m^3^ (69 ppb), and (**E**) 118 µg/m^3^ (96 ppb).

Figure 3 shows some typical fluorescence signals obtained with various gaseous formaldehyde concentrations ranging between 12 and 118 µg/m3 for given liquid and gas flow rates of 17 µL/min and 250 NmL/min, respectively. With the photomultiplier gain set at 40%, the fluorescence signal remained below the saturation value of 2.1 × 106 a.u., regardless of the formaldehyde concentrations. Prior to each measurement performed with a known gas concentration of formaldehyde, pure air was injected around the microporous tube to obtain a reference signal called the baseline or blank. The joint use of pure air and pure acetyl acetone solution free of formaldehyde for a few minutes allowed for a quick renewal of the interface and purge of the entire device. The fluorescence signal then fell back to its baseline level. No significant baseline deviation was observed during the duration of the experiments. The net average height of the fluorescence signal, corresponding to the formaldehyde concentration, was calculated by subtracting the baseline value from the raw height of the fluorescence signal. The error on this resulting signal was calculated to be two times the standard deviation. 

### 3.1. Influence of the Microporous Tube Length

The geometry and the size of the porous membrane can strongly affect the gas uptake, since both modify the total area of the interface. Here, the effect of the microporous tube length was studied under the same experimental conditions in the range 3.2–27.5 cm with 7 tubes, i.e., 3.2, 5.3, 7.5, 10.5, 13.0, 19.5, and 27.5 cm. The formaldehyde concentrations were set to 48.0 μg/m^3^ (39.3 ppb), corresponding to a medium indoor air level. Gas and liquid flow rates were fixed to 250 NmL/min and 17 µL/min, respectively.

**Figure 4:**
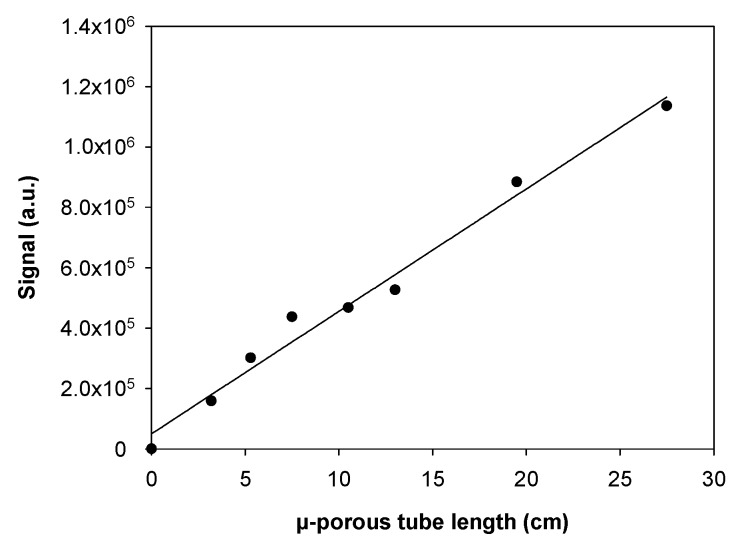
Influence of the microporous tube length in the range 3.2–27.5 cm on the fluorescence signal with a formaldehyde concentration of 48.0 µg/m^3^ (39.3 ppb), a photomultiplier gain set at 38% (saturation at 1.48 × 10^6^ a.u.), a liquid flow rate of 17 µL/min, and a gas flow rate of 250 NmL/min. There are vertical error bars corresponding to two times the standard deviation, but they are too small to be visible.

As shown in Figure 4, the fluorescence signal increased linearly with the microporous tube length up to 27.5 cm. Such an observation supported the assumption of the establishment of a dynamic equilibrium between the gas and liquid phases, where gas–liquid contact time influenced the gas uptake yield. This optimization tended to show that porous tube length could be modified according to the targeted concentration ranges measured, where a shorter tube could be used for highly polluted environments. Conversely, a longer tube will be better suited to quantify low airborne formaldehyde levels but will increase the response time of the analytical instrument.

In the framework of this study, the length of 10.0 cm seemed to be a good compromise to trap a significant amount of formaldehyde into the aqueous solution of acetylacetone without excessively increasing the response time of the instrument. Indeed, the internal volume of this porous tube was about 25.4 µL/cm, implying a renewal time of about 15 min at 17 µL/min with a 10-cm tube length. Consequently, this length was chosen for the following experiments. 

### 3.2. Influence of the Liquid Flow Rate

The liquid flow rate may influence the “pumping” effect through the porous membrane, modifying the gas uptake efficiency. This potential effect was investigated under the same experimental conditions by varying the liquid flow rate in the range 9–33 µL/min. Based on the results detailed in the previous section, the microporous tube length was fixed to 10.0 cm. The gas flow rate was equal to 250 NmL/min, while the gaseous formaldehyde concentration was 48.0 µg/m^3^ (39.3 ppb).

**Figure 5:**
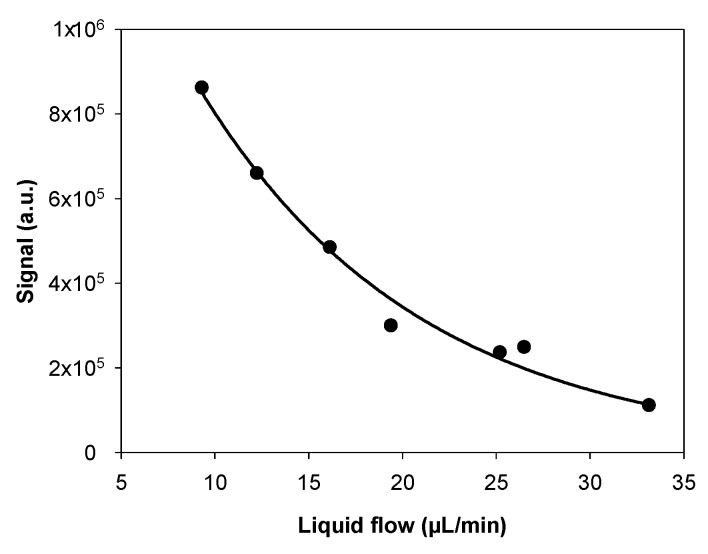
Influence of the liquid flow rate in the range 9–33 µL/min on the fluorescence signal with a formaldehyde concentration of 48.0 µg/m^3^ (39.3 ppb), a gas flow rate of 250 NmL/min, and a 10.0-cm microporous tube length. The photomultiplier gain was set at 34% (saturation at 1.48 × 10^6^ a.u.). There are vertical error bars that correspond to two times the standard deviation, but they are too small to be visible.

The results are displayed in Figure 5 where it is observed that the fluorescence signal decreased significantly when the liquid flow rate varied from 9 to 33 µL/min. In this range, the liquid flow rate was always sufficiently low to have a sufficient residential time in the oven to fully convert formaldehyde into DDL. Similarly, for the observations made for the influence of the microporous tube length, these experimental results confirmed that a dynamic equilibrium between gas and liquid phases was established and strongly depended on the gas–liquid contact time, which thus drove the efficiency of the gaseous formaldehyde uptake into the aqueous phase. In order to simultaneously minimize the response time and limit the reagent consumption, the liquid flow rate was fixed at 17 µL/min for the following experiments, with this value being a compromise between a rapid analytical response, an enhanced signal of fluorescence, and an acceptable reagent consumption. 

### 3.3. Influence of the Gas Flow Rate

The gas flow rate could modify the gaseous formaldehyde concentration at the porous interface by avoiding the scarcity of formaldehyde molecules near the exchange porous surface, which could be due to the rapid mass transfer to the aqueous solution. For this reason, the gas flow rate was varied in the range 50–300 NmL/min, whereas all the other parameters were maintained as follows: microporous tube length of 10.0 cm, liquid flow rate of 17 µL/min, and gaseous formaldehyde concentration of 84.6 µg/m^3^.

**Figure 6:**
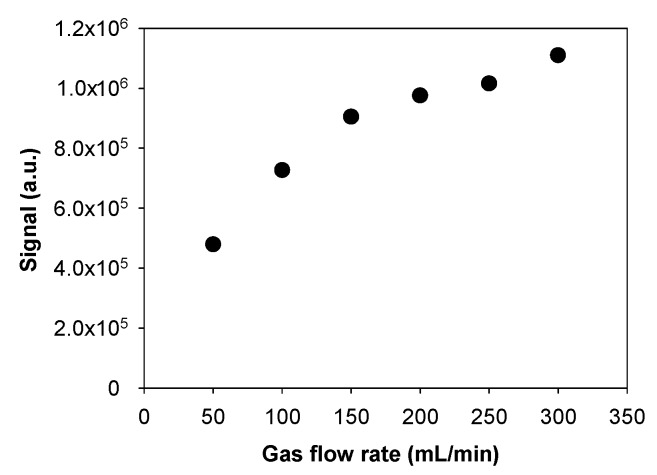
Influence of the gas flow rate in the range 50–300 NmL/min on the fluorescence signal with a formaldehyde concentration of 84.6 µg/m^3^ (69 ppb), a photomultiplier gain set at 50% (saturation at 2.07 × 10^6^ a.u.), a liquid flow rate of 17 µL/min, and a 10.0-cm microporous tube length cm. There are signal error bars that correspond to two times the standard deviation, but they are too small to be visible.

Figure 6 presents the results obtained and indicates that the fluorescence signal, and thus the formaldehyde uptake efficiency, strongly increased from 4.8 × 10^5^ to 11.1 × 10^5^ a.u. when the gas flow rate went up from 50 to 300 NmL/min. However, a plateau was not reached, suggesting that higher flow rates could improve the gas transfer until the point where the gas phase concentration near the porous surface would be kept constant such that the renewal of the gas mixture at the interface could be the kinetically limiting process while its uptake into the liquid phase itself where the gas goes through the membrane appears to be fast enough. 

### 3.4. Influence of Gaseous Formaldehyde Concentrations

This section demonstrates that the fluorescence signal was proportional to the gaseous concentration of formaldehyde when the experimental conditions were optimal. Indeed, an analytical device needs to be calibrated in the optimized conditions to give relevant measurements in the field.

Regarding the potential impact of the gas flow rate on the uptake efficiency, as demonstrated in the previous section, several calibrations curves were performed at different gas flow rates, i.e., 20, 60, 100, and 250 NmL/min. The gaseous formaldehyde concentrations varied in the range 0–118 µg/m^3^.

**Figure 7:**
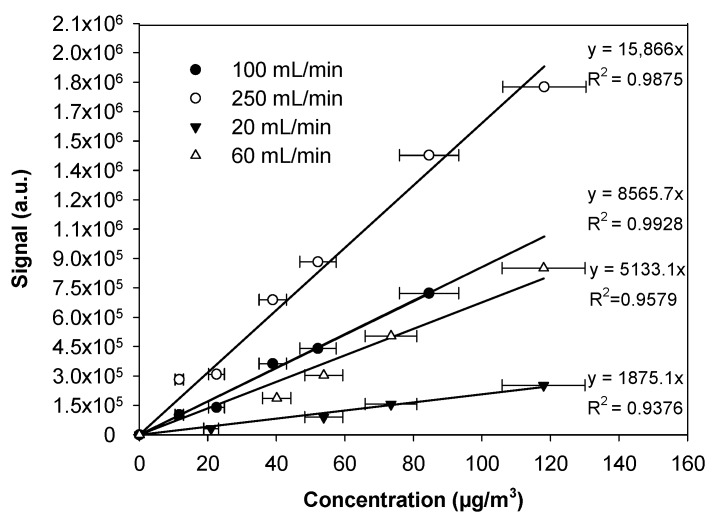
Calibration curve representing the fluorescence signal versus gaseous formaldehyde concentration in the range 0–118 µg/m^3^ (0–96 ppb) with a liquid flow rate of 17 µL/min, a photomultiplier gain set at 50% (saturation at 2.07 × 10^6^ a.u.), and a 10-cm microporous tube length. There are vertical error bars correspond to two times the standard deviation, but they are too small to be visible. The quoted horizontal error bars were calculated from the uncertainties of the mass flow controllers, the sampling gas, and the HPLC analysis of 2,4-dinitrophenylhydrazine (DNPH) tubes.

For all the gas flow rates investigated, the fluorescence signal increased linearly with the increase of the gaseous formaldehyde concentration, as displayed in Figure 7. This indicated that the formaldehyde amount trapped into the solution was perfectly proportional to that in the gas phase, regardless of the gas concentration and the gas flow rate. However, the slope of the linear plot increased from 1875 to 15,866 m^3^/μg when the gas flow rate varied between 20 and 250 mL/min. Even when comparing the highest gas flow rates, the slope of the linear fit at 100 NmL/min was approximately 40% lower than that obtained at 250 NmL/min.

A higher flow rate avoided the rarefaction of formaldehyde molecules near the microporous interface and minimized the establishment of a concentration gradient by quickly regenerating the gaseous mixture close to the interface. These results demonstrated that it was necessary to have an effective renewal of the gas concentration near the gas–liquid interface, regardless of the technical solution used, i.e., the use of a gas pump or a set of efficient fans. 

### 3.5. Analytical Performances

From the linear calibrations obtained at the highest gas flow rates of 100 and 250 NmL/min, the sensitivity of the microdevice could be evaluated from the signal intensity obtained at the lowest gaseous formaldehyde concentrations investigated, i.e., 11.2 (9.1 ppb) and 11.7 µg/m^3^ (9.5 ppb), respectively, and the experimental determination of the signal-to-noise ratio (S/N). Considering that the optimal gas flow rate for the gaseous formaldehyde uptake was 250 mL/min, the theoretical detection and quantification limits were estimated to be 0.13 μg/m^3^ (0.11 ppb) (S/N = 3) and 0.43 μg/m^3^ (0.35 ppb) (S/N = 10), respectively. The analytical prototype was slightly less sensitive at 100 mL/min, with values of 0.22 and 0.73 μg/m^3^ for the limits of detection and quantification, respectively.

In addition, the temporal resolution of 2 s and the response time of 15 min with a 10-cm microporous tube length allowed for the near real-time monitoring of airborne formaldehyde.

Furthermore, the experiments were carried out for 6 months with the same microporous tube sample that was 10 cm in length, showing that its lifetime was satisfactory.

### 3.6. Preliminary Integration in a Box Equipped with Fans

The possibility to replace the gas pump with fans was tested by placing the microporous tube in a box (20 cm × 25 cm × 15 cm) equipped with two fans sucking the ambient air in at the front, and two grids on the back sides to ensure the air extraction. The microporous tube was placed horizontally, and therefore vertically to the air stream generated, but not in a near proximity to the fans.

According to the observations made in the present work, the fluorescence signal increased depending on the environment in which the prototype was placed. Thus, with clean air, the signal was at the baseline level, i.e., around 46,800 µV, where this signal was obtained with an enhanced version of the data acquisition. In outdoor air, with a concentration of formaldehyde close to 6 μg/m^3^, the signal was already significantly greater and reached 95,000 µV (8030 µV m^3^/µg after subtracting the background). In polluted and confined indoor air where the formaldehyde level was of the order of 40 μg/m^3^, the signal increased again and reached 325,000 µV, i.e. 6960 µV m^3^/µg once the background was subtracted.

If the signals were perfectly stable, the signals were not found to be fully proportional to the gas phase concentration. From the signal measured at 6 µg/m^3^, and considering a noise of about 1000 µV, the detection limit was only of the order of 0.4 μg/m^3^. This lower sensitivity and nonlinear response could be explained using two main reasons:

- The selected fans were undersized; ideally, the linear gas velocities achieved should be almost identical to those used earlier in this study.
- The air flow around the microporous tube needed to be optimized by modeling, which could determine the optimal design for optimal air renewal near the microporous tube.

## 4. Conclusions

In this work, a microdevice was developed and the key parameters were optimized to obtain a linear response as a function of the gaseous formaldehyde concentration in the range 10–120 µg/m^3^, representative of the concentrations measured in indoor air.

The results obtained demonstrate the rarefaction of gaseous formaldehyde concentration at the microporous interface and the establishment of a concentration gradient due to the uptake into the solution. This key point needs to be considered for further prototype enhancement. Indeed, at a low turnover of the gas mixture close to the interface, there was clearly a kinetic limitation to an efficient gas–liquid transfer that could be explained by a slower gas diffusion compared to the uptake process itself. At a higher turnover of the gas mixture, this effect became limited.

This new analytical method allowed for the quantification of formaldehyde in near real-time. With a theoretical limit of detection (LOD) equal to 0.13–0.4 μg/m^3^, its very good sensitivity was enough to measure typical concentrations in both indoor and outdoor air. This LOD was also consistent with the upcoming French regulation limiting formaldehyde levels at 10 µg/m^3^ in 2023 in indoor air [7].

In the future, the new microdevice could operate in a fully passive sampling mode if the pump and the housing are removed and replaced by a set of fans to ensure the renewal of the gas mixture around the microporous interface at a sufficient gas flow rate and a subsequent gas velocity. Indeed, if the preliminary tests performed with two fans to replace the set of a costly mass flow controller and the noisy gas pump are encouraging, then further improvements are possible. With the help of modelling, such an implementation could lead to a reliable, quieter, and less-expensive device that is suitable for the monitoring of indoor air quality at a large-scale.
